# Irreversible Structural Changes of Copper Hexacyanoferrate Used as a Cathode in Zn‐Ion Batteries

**DOI:** 10.1002/chem.201905384

**Published:** 2020-02-25

**Authors:** Joohyun Lim, Ghoncheh Kasiri, Rajib Sahu, Kevin Schweinar, Katharina Hengge, Dierk Raabe, Fabio La Mantia, Christina Scheu

**Affiliations:** ^1^ Max-Planck Institut für Eisenforschung GmbH Max-Planck-Straße 1 40237 Düsseldorf Germany; ^2^ Universität Bremen, Energiespeicher- und Energiewandlersysteme Bibliothekstr. 1 28359 Bremen Germany

**Keywords:** copper hexacyanoferrate, degradation, electron energy loss spectroscopy, electron microscopy, energy conversion

## Abstract

The structural changes of copper hexacyanoferrate (CuHCF), a Prussian blue analogue, which occur when used as a cathode in an aqueous Zn‐ion battery, are investigated using electron microscopy techniques. The evolution of Zn_*x*_Cu_1−*x*_HCF phases possessing wire and cubic morphologies from initial CuHCF nanoparticles are monitored after hundreds of cycles. Irreversible introduction of Zn ions to CuHCF is revealed locally using scanning transmission electron microscopy. A substitution mechanism is proposed to explain the increasing Zn content within the cathode material while simultaneously the Cu content is lowered during Zn‐ion battery cycling. The present study demonstrates that the irreversible introduction of Zn ions is responsible for the decreasing Zn ion capacity of the CuHCF cathode in high electrolyte concentration.

## Graphical abstract

**The morphology, composition,** oxidation state, and crystal structure changes occurring in copper hexacyanoferrate‐based cathodes in aqueous Zn‐ion batteries are investigated to understand degradation of the cathodes after few hundred cycles. Electron microscopy reveals the substitution of Cu ions by Zn ions in the lattice resulting in the formation of wires and cubes from the initial copper hexacyanoferrate nanoparticles.

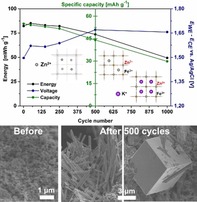

Charging/discharging metal ions is the key process for the storage of electric energy in the form of chemical energy in various metal‐ion batteries. This process is based on the redox reaction of electrode materials, accompanied by insertion and desertion of the metal cations, respectively.^1^ The reversibility of the reaction determines the long‐term stability and efficiency of metal‐ion batteries. The inserting and desertion process result in crystal distortion and phase transformation of electrode materials with varying lattice parameters and huge volume changes.^1^, ^2^ Dissolution of the electrodes, subsequent release of the chemical species in the electrolyte, and their re‐deposition on the opposite electrode, limit the long‐term use.^3^ The understanding of the overall reaction mechanism of metal‐ion battery process is inevitable to develop electrode materials with improved stability.

Prussian blue analogues (PBAs) are polynuclear transition metal cyanides, written as AM_α_[M_β_(CN)_6_]⋅*x* H_2_O, where A represents a monovalent metal cation, M_α_ is a high‐spin transition metal ion in M_α_N_6_ octahedra, and M_β_ is a low‐spin transition metal ion in M_β_C_6_ octahedra.^4^ PBAs have been considered promising active materials for various metal‐ion batteries because of their capability to reversibly insert and desert several metal ions.^5^ The robust and large 3D channel framework in PBAs allows the intercalation of even divalent Zn ions in aqueous electrolyte, which is cheap, safe, and potentially applicable for grid‐scale energy storage systems.^6^ The efficiency and stability of PBAs can be controlled by the choice of the metal species, the number and distribution of M_α_, M_β_, and M(CN)_6_ vacancies as well as interstitial water molecules.^5^, ^7^ This allows to design new Zn‐ion battery (ZIB) electrodes with superior performance which might overcome the limitations of vanadium oxide‐ and manganese dioxide‐based cathodes suffering from low electric conductivity and morphological‐ and structural changes during ZIB operation.^8^ Recently, an aqueous ZIB based on copper hexacyanoferrate (CuHCF), a PBA, showed promising specific energy and power density comparable to the ones of organic cells based on Li_4_Ti_5_O_12_ and LiFePO_4_.^9^ The large cavity in the 8c site (1/4 , 1/4 , 1/4 ) in CuHCF (interstitial sites) can be used as highly reversible Zn ion storage site with charge compensation given by Fe^3+/2+^ redox couple.

However, CuHCF is suffering from decreasing Zn ion capacity after hundreds cycles,^10^ without any obvious cathode dissolution or Zn dendrite formation.9a, 10 This indicates that the Fe^3+/2+^ redox couple for Zn ion storage is far from being ideal and there are most likely several factors limiting the long‐term stability. The degradation of CuHCF electrodes is more severe for high electrolyte concentration.^10^, ^11^ Even though X‐ray diffraction (XRD) revealed phase changes of CuHCF during cycling, the underlying mechanism and its relation to decreasing Zn ion capacity are still not clear.^12^ Therefore, detailed structural investigation is essential. Herein we report the morphology, composition, oxidation state, and crystal structure changes occurring in CuHCF‐based cathodes in aqueous ZIB. With the help of electron microscopy techniques, we were able to obtain the key information from each individual feature formed during cycling.

Crystalline CuHCF nanoparticles were synthesized by a controlled co‐precipitation method.^13^ The particle size of as‐prepared nanoparticles is less than 100 nm, as shown in transmission electron microscopy (TEM) and scanning TEM (STEM) images (Figure S1a and b). As illustrated in powder XRD and electron diffraction patterns (Figure S1c and d), CuHCF nanoparticles possess a face‐centered cubic crystal structure. The performance of this material is verified by applying the CuHCF nanoparticles as ZIB cathode in 100 mm of aqueous ZnSO_4_ electrolyte. Specific energy and capacity fading of the cathode after 250 cycles, accompanied by changes in the average voltage and morphology were observed (Figure 1 a and Figure S2). Although specific energy and capacity steadily decrease after 250 cycles, the electrochemical reaction potential of cathode, which is represented as *E*_WE_ *–E*_CE_, increases first and then remains constant after 500 cycles. This indicates that the main (de‐)intercalation reaction of Zn ions in the cathode after 500 cycles is different than before. Scanning electron microscopy (SEM) images from cathode surfaces cycled between 0 to 1000 times are presented with the highlighted images for 0, 500, and 1000 cycles in Figure 1 b and c. The CuHCF cathode surface after immersion in the electrolyte for 3 days but before ZIB cycling (0 cycle) reveals the same size and shape of the CuHCF nanoparticles as the nanoparticles in the initial powder. After 50, 150, and 250 cylcles, the surface of cathodes still shows similar morphological features as the original one with a small amount of additional plate and rod‐shaped particles. After 500 cycles, a higher amount of micron‐sized wire and cube‐shaped morphologies as well as initial CuHCF nanoparticles are observed, indicating morphological transformation of CuHCF cathode via reaction with Zn ions. Wires and cubes having additional facetted crystalline features on the main cube are found on the cathode surface after 1000 cycles.

**Figure 1:**
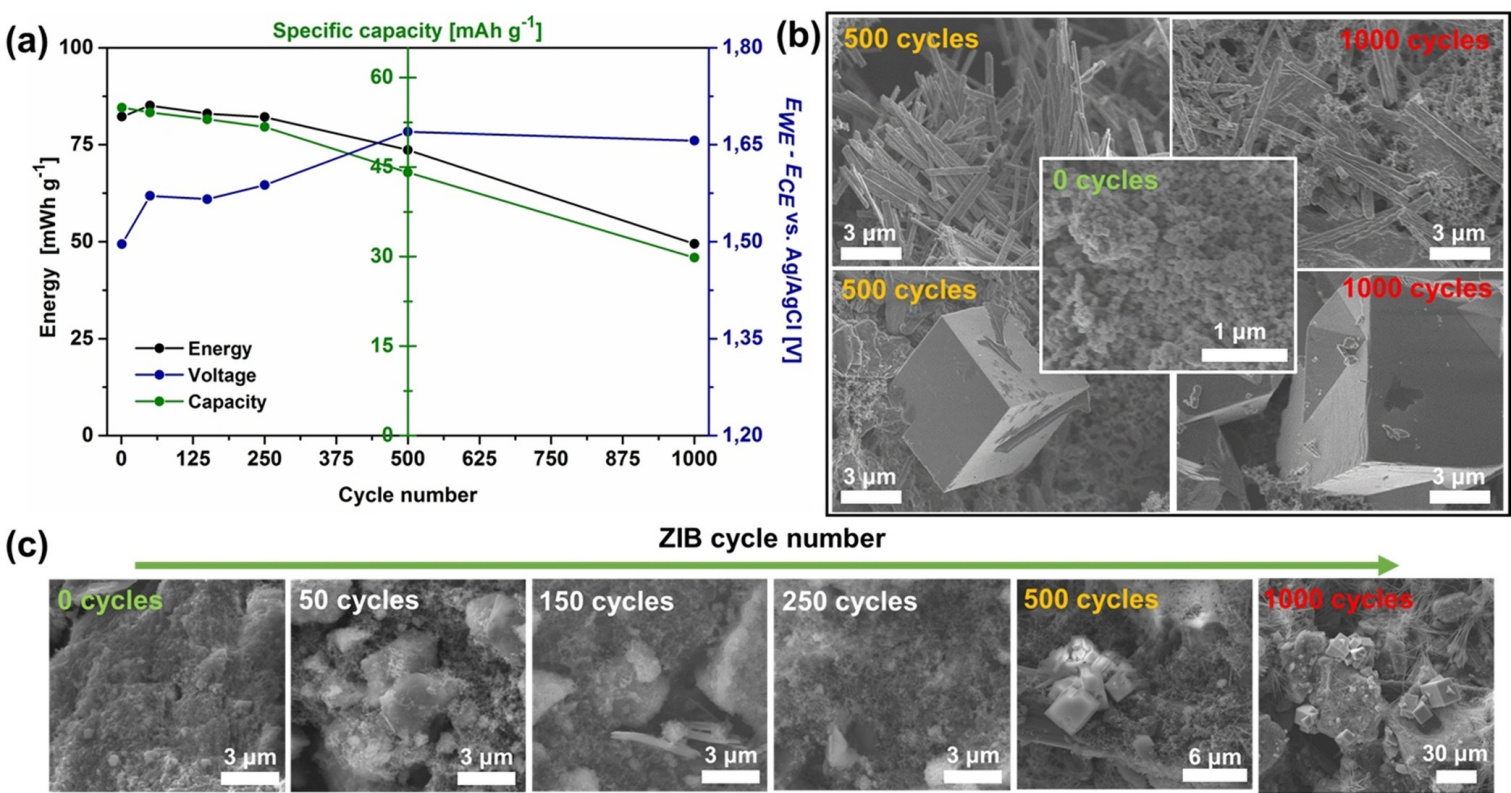
(a) Cycle performance of CuHCF cathode in 100 mm ZnSO_4_. (b) SEM images of wire and cube morphologies which form after 500 and 1000 cycles in comparison to the initial cathode surface. (c) SEM images of the cathode after 0, 50, 150, 250, 500, and 1000 cycles.

As the ZIB capacity is maintained up to 250 cycles where most part of the cathode surface is still composed of the initial nanoparticles and decreases afterward, the increasing amount of the wire and cube shaped structure is related to the ZIB performance. The different electrochemical reaction behavior of wire and cube structures compared to initial nanoparticles can be the main reason for decreasing Zn ion capacity up to 500 cycles. The main morphological changes occur within 500 cycles and the size of the wire and cube structures increases afterward, resulting in the decrease of the Zn ion capacity while *E*_WE_ *–E*_CE_ remains constant.

Different chemical compositions in each morphology are monitored using energy dispersive X‐ray spectroscopy (EDS). SEM‐EDS (Table S1) reveals that the relative amount of Cu in the cathode surface area consisting mainly of nanoparticles decreases with the number of cycles, while the Zn content is increasing. The Fe content remains similar. Wire and cube morphologies also have a lower amount of Cu and a higher amount of Zn compared to the initial CuHCF nanoparticles. The presence of Zn even after the electrochemical desertion of Zn ions indicates that this process is not perfectly reversible or Zn ions are not only located in the interstitial sites but also in the cubic lattice. To verify the chemical composition locally, STEM‐EDS is conducted (Figure 2 a–c and Table 1).

**Figure 2:**
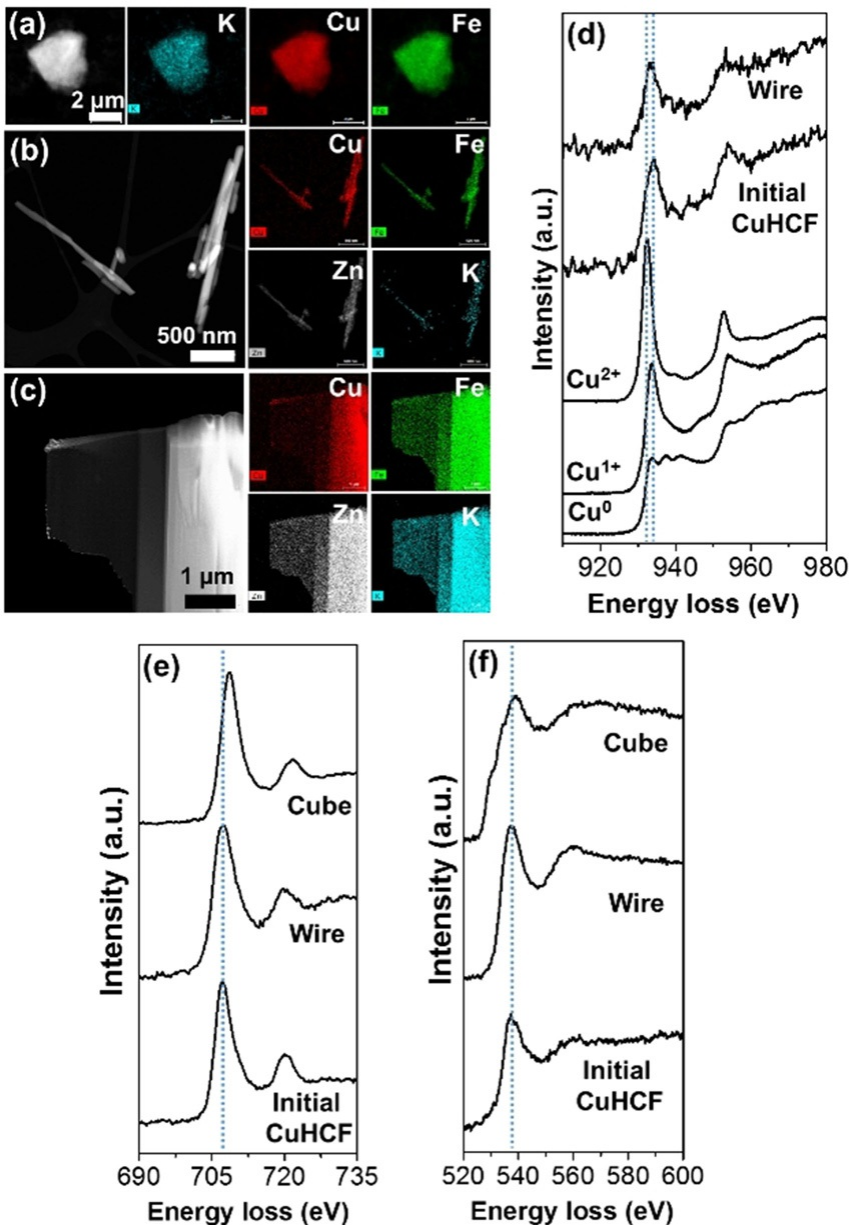
STEM images and STEM‐EDS mappings of (a) initial CuHCF nanoparticles, (b) wire, and (c) cube morphologies. Core‐loss EELS of (d) Cu‐L_2,3_,* (e) Fe‐L_2,3_ and (f) O‐K edges. * Reference EELS data representing Cu^0^, Cu^1+^, and Cu^2+^ are taken from ref. [18].

**Table 1:** Chemical content revealed from STEM‐EDS, Fe I(L_3_)/I(L_2_) edge intensity ratio obtained from EELS spectra.

CuHCF	Initial nanoparticles	Wire	Cube
Cu (at.%)	60	23	13
Fe (at.%)	37	28	28
Zn (at.%)	0	47	32
K (at.%)	3	2	27
Cu/Fe	1.6	0.82	0.46
I(L_3_)/I(L_2_)_Fe_	3.9	4.4	5.6

The TEM sample of the initial CuHCF nanoparticles and the wires obtained after 1000 cycles are prepared from a colloidal suspension and drop casting on a TEM grid. For the micrometer sized cube features, focused ion beam (FIB) is used to obtain thin TEM lamella (Figure S3). Wires and cubes from the 1000 cycled cathode have a lower Cu to Fe ratio compared to the initial CuHCF nanoparticles while the Zn content increases. Regarding decreasing amount of Cu, Zn ions can substitute Cu and occupy the specific sites. Noticeably, only cubes contain a high amount of K ions, implying a specific role of K ions in the development of a cube rather than wire. One possibility is the presence of cubic potassium zinc cyanide structure locally, stabilizing the overall cubic morphology.^14^

To investigate the substitution mechanism occurring in CuHCF upon insertion of Zn ions, electron energy loss spectra (EELS) were obtained in STEM mode. Figure 2 d presents the Cu‐L_2,3_ edge from initial CuHCF nanoparticles and a wire from the 1000 cycled cathode. Both of them show two white‐lines, indicating the presence of cationic Cu. The energy loss values of the Cu‐L_2.3_ edge from the wire is assigned to a mixture of mono‐ and divalent Cu, revealing that the Cu^2+/1+^ redox couple also works during cation (de)intercalation. The Cu^2+/1+^ as well as Fe^3+/2+^ redox couple can be the main reason of the morphological change and phase separation of CuHCF in aqueous ZIB system.^15^ The dual redox charge compensation mechanism of CuHCF is understood by two electrons confined to a cyanide‐bridged Cu and Fe unit due to the strong association with a divalent cation. This results in electron filling of Fe t_2g_ as lowest unoccupied molecular orbital (LUMO) and that of Cu e_g_ as next LUMO.^16^ Even Cu ions in initial CuHCF nanoparticles seem to be also mono‐ and divalent as indicated by the shape of Cu‐L_3_ line. This could stem from the intercalation of K^+^ and acceptance of electrons simultaneously from the cyanide group which is an electron donor.^17^ The Cu‐L_2,3_ edge from the cubic morphology could not be obtained due to the low amount of Cu and high sensitivity of thin TEM lamella under electron beam bombardment.

All samples possess two distinct L_3_ and L_2_ peaks at the Fe‐L_2,3_ edge as shown in Figure 2 e. The higher the intensity ratio of the two peaks (I(L_3_)/I(L_2_)_Fe_), the higher the oxidation state of Fe species.^19^ (Table 1). The increasing I(L_3_)/I(L_2_)_Fe_ value indicates that the content of Fe^3+^ ions increases from the initial CuHCF nanoparticles via the wire to the cube, suggesting a decreasing reduction of Fe^3+^ to Fe^2+^ with increasing Zn ion content. First, the presence of Fe^2+^ already in the initial CuHCF nanoparticles can be understood by the reduction of Fe^3+^ to Fe^2+^ because of K^+^ intercalation, which was observed by Mössbauer spectroscopy in an earlier study.^20^ The higher amount of Zn ions in the wire and cube, however, does not increase but decrease the amount of Fe^2+^. This reveals that the Zn ions here are not at interstitial sites but substitute Cu ions in case of the wire and cube structures. We propose that this substitution of Cu ions by electrochemically inactive Zn ions^21^ leads to a phase transformation and is the main reason of capacity degradation of CuHCF cathode in aqueous ZIB. This is because CuHCF loses the Cu^2+/1+^ redox couple which also plays a role for charge compensating of divalent metal cation during intercalation,^15^, ^22^ and consequently a lower Fe^3+/2+^ redox efficiency is present in the wire and cubic morphologies. The severe capacity loss of the cathode is found after around 500 cycles (Figure 1), where many wires and cubes are formed. Figure S4 shows the formation of new cathodic and anodic peaks in the differential charge curve arising after ≈500 cycles, indicating that the Zn ion (de)intercalation chemistry of CuHCF cathode changes. CuHCF seems to transform in a form of Zn_*x*_Cu_1−*x*_HCF. To explain the additional insertion of Zn ions from the interstitial site to the substitutional site, Zn ion hopping to Fe(CN)_6_ vacancy sites was suggested using synchrotron XRD measurements.^22^ However, this hopping mechanism was observed only at the first ZIB cycle without Cu ion loss and cannot be related to the morphology change and ZIB capacity decrease here. After Zn ions are introduced in the interstitial sites, a higher concentration of Zn ions in the electrolyte together with an external bias gives CuHCF a better chance to uptake more Zn ions to the substitutional site. Divalent Zn ions interact with the nitrogen of the cyanide group rather than with carbon due to their low‐spin electron configuration. In the end, new ‐Zn‐*N*‐ bonds can form removing Cu ions from the cubic CuHCF framework. Because this reaction slowly proceeds during long‐term ZIB cycles, small and irregular shaped initial CuHCF nanoparticles (kinetic product) transform to bigger wire and cubic morphologies with higher crystallinity (thermodynamic product).^23^

The nanoparticles, wires, and cubes possess similar oxygen K‐edges in the EELS spectra with the first maximum centered at ≈537 eV, a value higher than that of most metal oxides (Figure 2 f). The O K‐edge can be assigned to coordinated‐, intercalated‐, or interstitial water molecules.4b, 24

The overall crystal structure change of the CuHCF cathode during ZIB cycle was investigated using XRD (Figure S5). The XRD patterns indicate changes already directly after immersing CuHCF cathode in the electrolyte even before the first ZIB cycle. A new peak at ≈2*θ*=27° visible as a shoulder and a sharp peak at ≈2*θ*=33° cannot be assigned. It seems that Zn ions are introduced to CuHCF and change the local crystal structure, regarding the fast cation and water exchange ability of CuHCF in electrolytes.^20^, ^22^ Other new peaks with higher intensity are found in 500‐ and 1000 cycled cathodes, which can be assigned to the (200), (220), (400), and (420) planes of the cubic ZnHCF phase and others to the (024), (116), (300), and (119) planes of the rhombohedral ZnHCF structure. Despite the decreasing peak intensity, the peaks of cubic CuHCF phase are still present in the 1000 cycled cathode. The XRD results demonstrate that the irreversible Zn ion substitution of Cu ions transforms the initial cubic CuHCF crystal structure to cubic Zn_*x*_Cu_1−*x*_HCF as a main phase possessing both Cu‐ and Zn‐rich crystalline regions and local rhombohedral Zn_*x*_Cu_1−*x*_HCF as a minor phase.

Initial CuHCF nanoparticles loose Cu ions during their transformation into Zn_*x*_Cu_1−*x*_HCF but very little metal cations are found in the electrolyte.^10^ This can be explained by the presence of two different Cu‐rich morphologies in the cathodes (Figure 3). The STEM image of a FIB lamella (Figure S6a) prepared from a 500 cycled cathode shows brighter particles ≈200 nm in size surrounded by dark appearing connected particles (Figure 3 a). These bright particles are assigned to a Cu‐rich phase without Fe ions based on STEM‐EDS, indicating phase separation from the initial CuHCF nanoparticles. Considering the oxygen EDS mapping, these particles could not be assigned to CuO_*x*_. It is assumed that Cu ions are rather well stabilized by cyanide bridges.^25^ The Cu‐rich particles are highly crystalline as shown in selected area electron diffraction (Figure 3 b) and possess a porous structure (Figure S6b–d). The overall presence of finer Cu‐rich area in the lamella is visualized using dark‐field TEM imaging in lower magnification (Figure 3 c). The other Cu‐rich phase is shown in Figure 3 d. These fine particles are prepared from the colloidal suspension of the 1000 cycled CuHCF cathode. Different from the Cu‐rich structure described above, Zn as well as Cu is detected here, indicating co‐ stabilized Cu‐ and Zn ions by cyanide bridging. Both Cu‐rich phases are formed by Cu ions released from the initial nanoparticles and are assumed to be inert towards Zn ion intercalation leading to performance loss. All morphologies over 500 cycles can be summarized as Equation (1) below:

(1) $$\mathrm{KCuFe}\left(\mathrm{CN}\right){}_{6}\to a\mathrm{KCuFe}\left(\mathrm{CN}\right){}_{6(\mathrm{initial}\;\mathrm{CuHCF}\;\mathrm{nanoparticels})}\;+\;b\mathrm{KCu}{}_{1-x}\mathrm{Zn}{}_{x}\mathrm{Fe}\left(\mathrm{CN}\right){}_{6(\mathrm{wires}\&\mathrm{cubes})}\;+\;c\mathrm{Cu}\left(\mathrm{CN}\right){}_{6(\mathrm{Cu}\text{-}\mathrm{rich}\;\mathrm{areas})}\;+\;d\mathrm{CuZn}\left(\mathrm{CN}\right){}_{6(\mathrm{Cu}\text{-}\mathrm{rich}\;\mathrm{areas})},\;\mathrm{where}\;a+b+c+d=1$$

**Figure 3:**
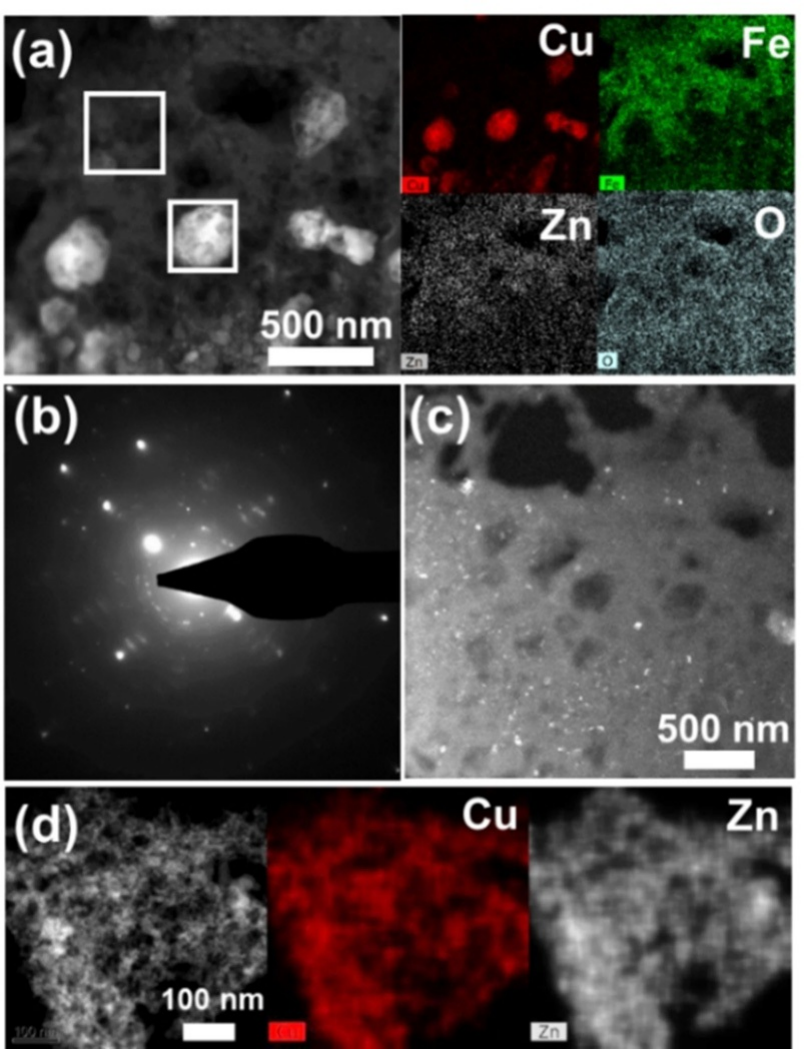
STEM images and STEM‐EDS mappings of Cu‐rich area in (a) 500 and (d) 1000 cycled cathodes. White boxes mark where STEM‐EDS is monitored. (b) Selected area diffraction patterns from bright area in (a) and (c) dark‐field image.

This work has shed new light into the structural change of CuHCF, and a previously proposed mechanisms can be corrected in order to take into account the release of Cu.^10^, ^12^ Substitution of Cu ions by Zn ions in substitutional as well as in interstitial sites induces formation of wires and cubes from the initial CuHCF nanoparticles. Simultaneously, Cu‐rich structures are formed by the released Cu ions, which are inactive towards Zn ion storage. In an earlier work, we observed that the best specific charge stability was obtained for ZnSO_4_ among other electrolytes such as ZnF_2_, Zn(ClO_4_)_2_, and Zn(NO_3_)_2_.^10^ Accordingly, we expect that the irreversible transformation for these electrolytes will occur already after even lower number of cycles. Since the electrochemical properties of PBAs are strongly dependent on their transition metal content and defects, it is evident that optimization of the material requires detailed structural studies using electron microscopy techniques.

## Conflict of interest

The authors declare no conflict of interest.

## Supporting information

As a service to our authors and readers, this journal provides supporting information supplied by the authors. Such materials are peer reviewed and may be re‐organized for online delivery, but are not copy‐edited or typeset. Technical support issues arising from supporting information (other than missing files) should be addressed to the authors.

Supplementary
