# Supplementary material for: Irreversible Structural Changes of Copper Hexacyanoferrate Used as a Cathode in Zn‐Ion Batteries
Source: Chemistry. 2020 Feb 25;26(22):4917–22. doi: 10.1002/chem.201905384 (PMC7187350; doi:10.1002/chem.201905384)
Supplement: Supplementary file 1 — Supplementary [file CHEM-26-4917-s001.pdf]

Chemistry–A European Journal

Supporting Information

Irreversible Structural Changes of Copper Hexacyanoferrate Used as a Cathode in Zn-Ion Batteries

Joohyun Lim,*[a] Ghoncheh Kasiri,[b] Rajib Sahu,[a] Kevin Schweinar,[a] Katharina Hengge,[a] Dierk Raabe,[a] Fabio La Mantia,[b] and Christina Scheu*[a]

Experimental Procedures

Synthesis of CuHCF

CuHCF nanoparticles were synthesized by means of a co-precipitation method.[1] 120 mL of 100 mM $Cu(NO_3)_2 \cdot 3H_2O$ (98-103%, Sigma-Aldrich) and 120 mL of 50 mM of $K_3Fe(CN)_6$ (≥99%, Sigma-Aldrich) were simultaneously added dropwise to 60 mL H_2O under vigorous stirring using a peristaltic pump at room temperature. The dripping was controlled using a pump with a flow rate of 0.1 mL min^{-1} for 2h. After finishing pumping, the resulting suspension was stirred for 30 minutes, followed by sonicating for 30 minutes. Thereafter, the suspension rested overnight. At last, the precipitate was centrifuged and washed with a solution containing 1 M KNO_3 (≥99%, Sigma-Aldrich) and 10 mM HNO_3 (≥65%, Sigma-Aldrich), followed by distilled water, and was dried at 60°C overnight. The fine powder was ground with a mortar and pestle.

Electrochemical measurement

In order to prepare the cathode for electrochemical tests, a slurry containing 80 wt% CuHCF nanoparticles, 2 wt% graphite (SFG6, Timcal), 9 wt% amorphous carbon (C65, Timcal) as a conductive additive, and 9 wt% polyvinylidene fluoride (PVDF, Solvay) binder solution in *N*-methylpyrrolidine (≥99.5%, Sigma-Aldrich, 25 mg mL^{-1}) was mixed via an Ultra-Turrax disperser. The slurry was stirred for 30 min at 4000 rpm. Afterwards, the slurry was painted on a carbon cloth current collector with a mass loading of ~10 mg per cm^2. For performing the electrochemical measurements a flooded three-electrode cell was used. The synthesized CuHCF nanoparticles were used as working electrode's active material, Zn foil (99.99%, Goodfellow) as counter electrode, Ag/AgCl (3 M KCl) as reference electrode, and 100 mM $ZnSO_4$ (≥99.9%, Sigma-Aldrich) solution as an aqueous electrolyte. The electrochemical measurements were performed using a BioLogic VMP3 instrument.

Characterization.

Scanning electron microscopy (SEM). SEM was performed to investigate the morphology change of the CuHCF cathode after a number of cycles under aqueous Zn-ion battery condition using a Zeiss 1540XB operated at 15 kV and a Zeiss Gemini 500 with in-lens detector operated at 2 kV. Energy dispersive X-ray spectroscopy (EDS) was done in SEM using a Zeiss 1540XB with a TSL Apollo XL Silicon Drift detector to monitor the chemical composition of the CuHCF cathodes.

(Scanning) Transmission electron microscopy ((S)TEM). (S)TEM was performed using a FEI 60-300 Titan Themis operated at 300 KV with a Cs-corrector for the probe forming lens. The chemical composition of CuHCF was confirmed by EDS in the STEM mode using a Bruker X-EDS detector. EELS data were acquired in the STEM mode using dual channel acquisition mode.[2] All spectra were corrected for channel to channel gain variations and dark current.[3] The background was subtracted using a standard power law. Post-edge background of Fe-L2,3 edge EELS were further removed with a double arctangent step function.[4] The $I(L_3)/I(L_2)_{Fe}$ values were obtained by integration over a 2 eV window.

Focused ion beam (FIB). TEM lamella were prepared using a FIB (ZeissNVision40 FIB microscope and FEI Helios NanoLab 600) integrated with a SEM. The lamella were obtained using the conventional lift out method.[5]

X-ray diffraction (XRD) was performed using a Rigaku MiniFlex 600 X-ray diffractometer with a step width of 0.02°, using Cu Kα radiation (λ = 1.5406 Å).

Results and Discussion

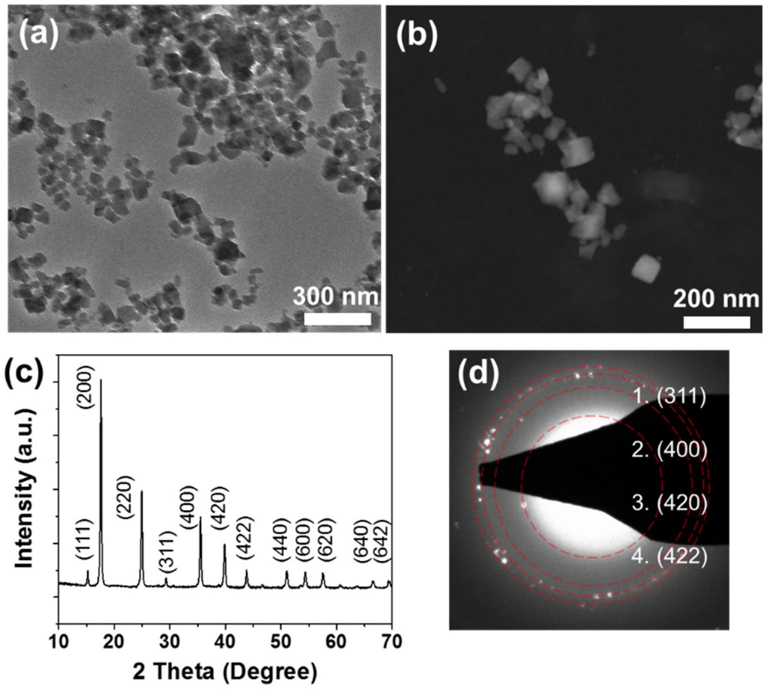

Figure S1. (a) TEM image, (b) STEM image, (c) XRD pattern, and (d) electron diffraction pattern of initial CuHCF nanoparticles.

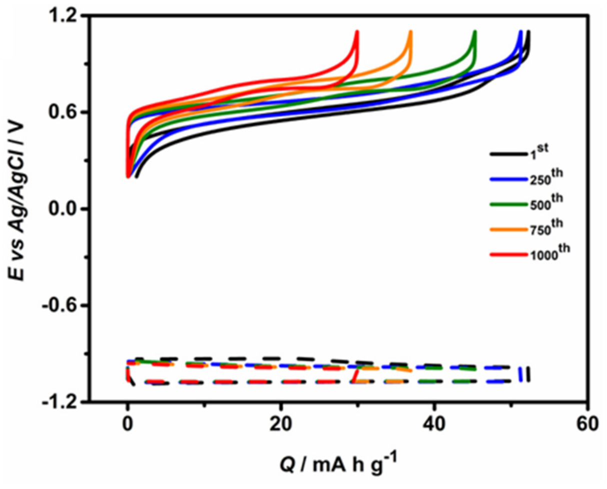

Figure S2. Potential profile at different numbers of cycles of CuHCF measured at a rate of 1 C in 100 mM $ZnSO_4$.

Table S1. Metal cation content of cycled cathodes using SEM-EDS.

	0 cycle	50 cycles		150 cycles	250 cycles	500 cycles			1000 cycles		
	Particle	Particle	Aggregated	Particle	Particle	Particle	Wire	Cube	Particle	Wire	Cube
Cu (at.%)	59±1	46±1	43.5±0.7	35.0±0.4	41.1±0.0	20±7	19.4±0.2	2±2	39.1±0.3	18.1±0.3	3±1
Fe (at.%)	39±1	36.4±0.2	30.2±0.4	33.0±0.1	35±1	40±13	33.8±0.2	35.1±0.6	34±1	32.8±0.6	33±15
Zn (at.%)	0.0	0.0	11.9±0.0	16.4±0.3	17±2	38±13	45.9±0.1	59±1	15.3±0.7	45.7±0.5	23±5
K (at.%)	2.5±0.2	18.1±0.7	14.3±0.3	15.6±0.8	7±3	2.0±0.7	1.0±0.1	4±2	12±2	3.4±0.9	40±12

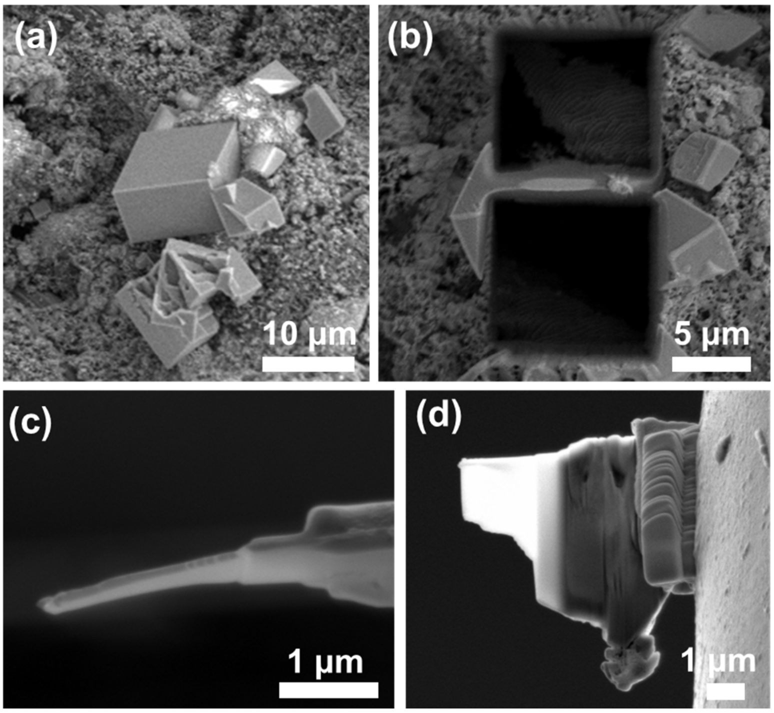

Figure S3. SEM images of (a) cube structure from 1000 cycled cathode and (b) cut out area. (c) FIB and (d) SEM images of final TEM lamellae of the cube sample.

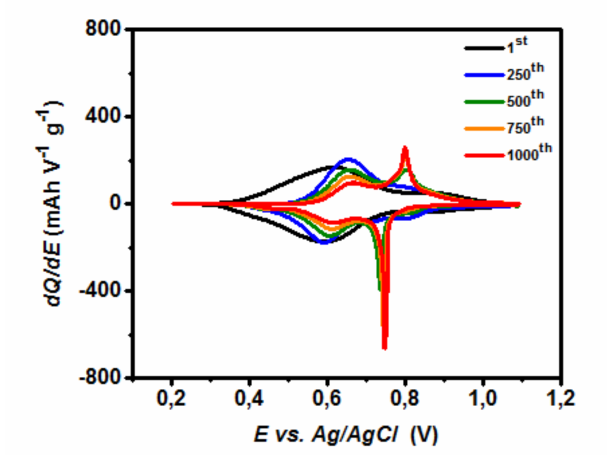

Figure S4. Differential specific charge profiles at different numbers of cycles of CuHCF measured at a rate of 1 C in 100 mM $ZnSO_4$.

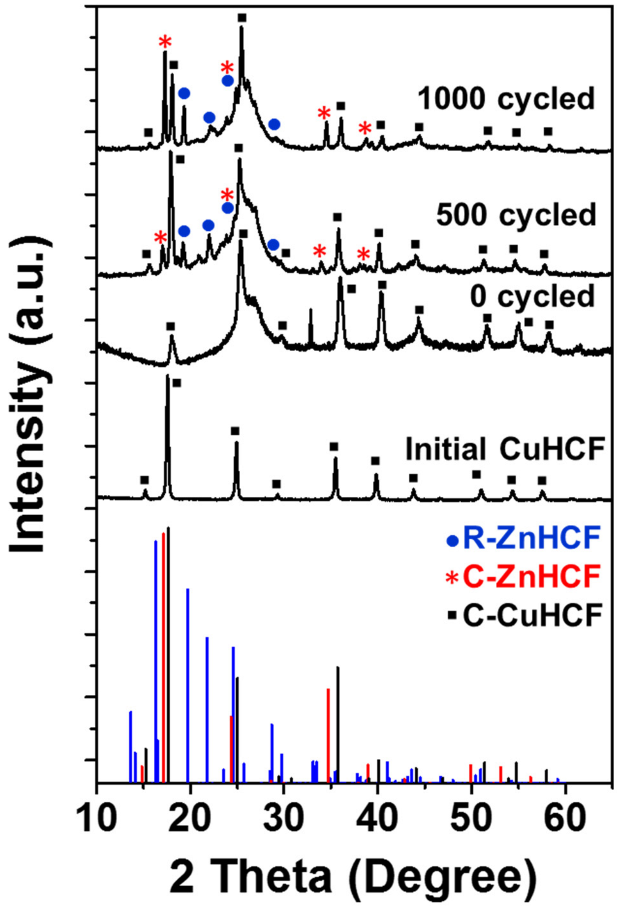

Figure S5. XRD patterns of the initial CuHCF nanoparticles, cathodes before cycle (0 cycles) but after being immersed in electrolyte, after 500 cycles, and after 1000 cycles. Black dot, red star, and blue circle indicate cubic CuHCF, cubic ZnHCF, and rhombohedral ZnHCF, respectively.

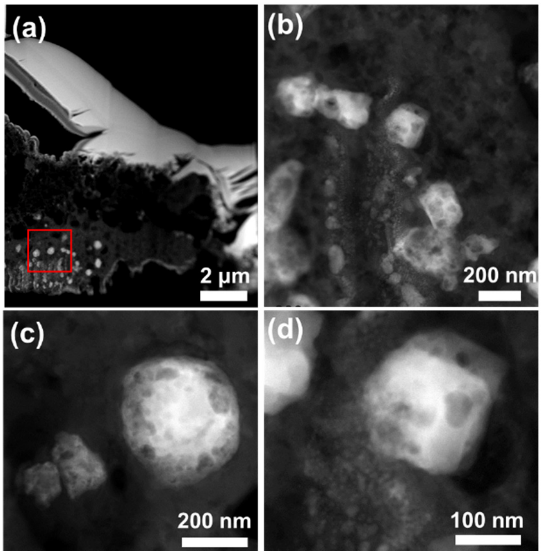

Figure S6. STEM image of (a) whole thin TEM lamellae from 500 cycled cathode and (b-d) bright Cu-rich structures. Red box presents the area where STEM-EDS is monitored.

References

[1] C. D. Wessells, R. A. Huggins, Y. Cui, *Nat. Commun.* **2011**, *2*, 550.

[2] J. Scott, P. J. Thomas, M. MacKenzie, S. McFadzean, J. Wilbrink, A. J. Craven, W. A. P. Nicholson, *Ultramicroscopy* **2008**, *108*, 1586-1594.

[3] R. F. Egerton, *Rep. Prog. Phys.* **2008**, *72*, 016502.

[4] L. Cavé, T. Al, D. Loomer, S. Cogswell, L. Weaver, *Micron* **2006**, *37*, 301-309.

[5] L. A. Giannuzzi, F. A. Stevie, *Micron* **1999**, *30*, 197-204.

Author Contributions

J.L. designed and carried out the electron microscopy experiments, analyzed the data, and wrote the manuscript. R.S., K.S., and K.H carried out a part of the electron microscopy experiments. G.K. conducted electrochemical experiments, synthesized the materials, and contributed to writing the manuscript. F.L.M. contributed to conception of the manuscript and data analysis. D.R contributed to data analysis. C.S. contributed to the conception and design of the experiment, analysis, and manuscript writing. All authors contributed and gave approval to the final version of the manuscript.
